# Health Tracts

**Published:** 1860-02

**Authors:** 


### HEALTH TRACTS.

THE Editor has been solicited at various times, by strangers as well as friends, to republish articles which have appeared from time to time in the *Journal of Health*. This has suggested a better plan, which is to make a selection of those which are of more universal application, and have them printed in the form of tracts for general distribution. The *fac-simile* of each is given in the following pages. Those of our subscribers who wish to exercise their benevolence in distributing them, can have them sent, assorted and post-paid, for twenty-five cents a hundred. The postage on a single tract is one cent, each piece of printed paper being liable to a separate postage, but when fifty or a hundred are sent, printed on one piece of paper, the postage is the same; hence they will be sent assorted in pamphlet form, and they can be easily divided. And as we desire to take care of number one while we are doing a good turn to number two, an advertisement of our publications will be found on the reverse of each tract; so being free-hearted, we don't charge for the advertisement, only for the advice on the opposite page. The Editor of the *Journal of Health* is a very generous person, sometimes, by fits and starts, pretty much according to the state of the weather, the stomach, and the money-market

